# Primary malignant melanoma of the esophagus: differentiation from esophageal squamous cell carcinoma and leiomyoma using dynamic contrast-enhanced CT findings

**DOI:** 10.1007/s00261-022-03556-8

**Published:** 2022-06-06

**Authors:** Yan-Jie Shi, Xin Yang, Shuo Yan, Xiao-Ting Li, Yi-Yuan Wei, Xiao-Yan Zhang, Ying-Shi Sun

**Affiliations:** 1 Key Laboratory of Carcinogenesis and Translational Research (Ministry of Education), Department of Radiology, Peking University Cancer Hospital & Institute, No. 52 Fu Cheng Road, Hai Dian District, Beijing, 100142 China; 2 Key Laboratory of Carcinogenesis and Translational Research (Ministry of Education), Department of Pathology, Peking University Cancer Hospital & Institute, No. 52 Fu Cheng Road, Hai Dian District, Beijing, 100142 China

**Keywords:** Esophagus, Melanoma, X-ray computed tomography, Esophageal cancer, Leiomyoma

## Abstract

**Purpose:**

This study aimed to summarize the computed tomography (CT) findings of PMME and differentiate it from esophageal SCC and leiomyoma using CT analysis.

**Methods:**

This was a retrospective study including 23 patients with PMME, 69 patients with SCC, and 21 patients with leiomyoma in our hospital. Qualitative CT morphological characteristics of each lesion included the location, tumor range, ulcer, enhanced pattern, and so on. For quantitative CT analysis, thickness, length and area of tumor, size of largest lymph node, number of metastatic lymph node, and CT value of tumor in plain, arterial, and delayed phases were measured. The associated factors for differentiating PMME from SCC and leiomyoma were examined with univariate and multivariate analysis. Receive operating characteristic curve (ROC) was used to determine the performance of CT models in discriminating PMME from SCC and leiomyoma.

**Results:**

The thickness, mean CT value in arterial phase, and range of tumor were the independent factors for diagnosing PMME from SCC. These parameters were used to establish a diagnostic CT model with area under the ROC (AUC) of 0.969, and accuracy of 90.2%. In pathology, interstitial vessels in PMME were more abundant than that of SCC, and the stromal fibrosis was more obvious in SCC. PMME commonly exhibited intraluminal expansively growth pattern and SCC often showed infiltrative pattern. The postcontrast attenuation difference in maximum CT attenuation value between plain and arterial phases was the independent factor for diagnosing PMME from leiomyoma. This parameter was applied to differentiate PMME from leiomyoma with AUC of 0.929 and accuracy of 86.4%.

**Conclusion:**

The qualitative and quantitative CT analysis had excellent performance for differentiating PMME from SCC and esophageal leiomyoma.

**Graphical abstract:**

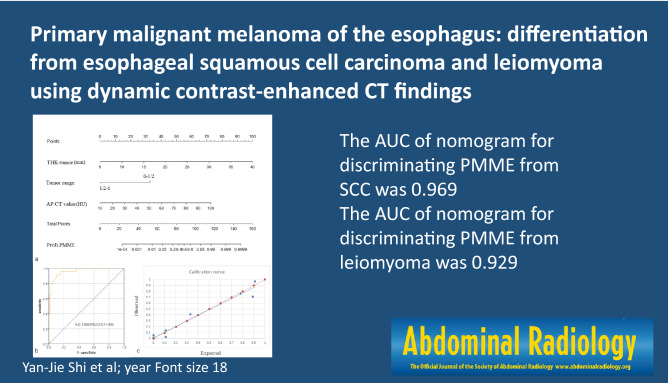

**Supplementary Information:**

The online version contains supplementary material available at 10.1007/s00261-022-03556-8.

## Introduction

Primary malignant melanoma of the esophagus (PMME) is extremely rare, accounting 0.1–0.5% of esophageal malignancies [1]. PMME has a highly aggressive biological behavior and its prognosis is poor, with 5-year overall survival of < 5% [2]. Approximately, 40–80% of newly diagnosed PMME patients have distant metastases [3]. The main treatment method of PMME is radical resection of tumor. Chen et al. found that early detection of the disease and radical resection of the tumor were critical for better survival of the PMME patients [4]. However, the risk recurrence is extremely high after an initial staging operation, and the interval between primary surgery and recurrence was only 4.5 months [5]. Other therapies include chemotherapy, chemoradiotherapy, endocrine therapy, and immunotherapy; the roles of these treatment strategies remain unclear [6]. Therefore, the identification of PMME before treatment is of important significance for clinical decision-making and contributes to determining an appropriate treatment strategy for PMME.

The preoperative diagnostic rate of PMME was low [4]. Pathological diagnosis of PMME is the gold standard; however, accurate PMME diagnosis before surgery is difficult even with endoscopic biopsy. Previous studies reported that 20% of patients were misdiagnosed as a poorly differentiated carcinoma due to lacking the characteristic dark surface and melanin granules [3, 7]. In addition, biopsy for PMME may increase the risk of dissemination or metastasis [7].

Since melanoma is thought to be arising from the basal layer of the epithelium and is covered by superficial epithelium, radiological appearance closely resembles non-epithelial tumors. Leiomyoma is the most common mesenchymal tumor of the esophagus [8]. Preoperative diagnosis of esophageal leiomyoma is often a challenge [9]. The use of the biopsy in the diagnosis is controversial. It may result in many complications such as infection, bleeding, and increasing rate of perforation [10]. In some cases, needle aspiration biopsy does not accurately identify the nature of the lesion [10].The most common imaging modalities, such as computed tomography (CT), PET–CT, and endoscopic ultrasound (EUS), play a crucial role in the diagnosis of esophageal disease [11–13]. PET–CT has been regarded as a cost-ineffective strategy and should not yet be used in routine clinical practice [14]. EUS is limited in evaluating some advanced tumors whose outer borders might be outside the field of view, especially stenotic tumors [15]. Therefore, chest CT was also inexpensive, easy to perform, reproducible, and could provide morphological and quantitative information of lesions and surrounding conditions. So, CT may provide useful information for diagnosing PMME and differentiating it from SCC and leiomyoma.

Because of the rarity of PMME, very few studies have investigated the radiological characteristics. Previous reports found that PMME was usually polypoid, intraluminal, and nonobstructive [16, 17]. However, the differential diagnosis of PMME based on both clinical and imaging manifestation is a challenge in clinical practice. So, in this study, we summarize the computed tomography (CT) findings of PMME and differentiate it from SCC and esophageal leiomyoma using qualitative and quantitative CT analysis.

## Materials and methods

This retrospective study was approved by our institutional review board, and a waiver of informed consent was remitted.

### Patients

**Fig. 1:**
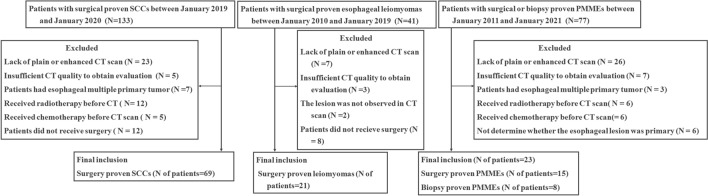
Patients flowchart

We searched for patients with surgical or biopsy-proven PMME at our hospital between January 2011 and January 2021, and 23 consecutive patients were identified. The following criteria were applied: (1) Patients with gastroscopy biopsy-proven or surgical proven PMME; (2) Patients performed chest enhanced CT examination; (3) Availability of diagnostic quality images for measuring lesions; (4) The primary site of melanoma was identified as esophagus. The exclusion criteria were as follows: (1) Patients had esophageal multiple primary tumor; (2) Patients received other treatment, such as chemotherapy or radiotherapy before CT scan; (3) Enhanced or plain chest CT data could not be obtained or the images could not be interpreted; (4) Presence of other primary sites of melanoma. In addition, 69 esophageal squamous cell carcinoma (SCC) patients who underwent surgery were included in this study. 21 patients with surgical proven leiomyoma at our hospital between January 2010 and January 2019 were identified. Finally, a total of 113 patients were obtained in this study. The complete patient enrollment process is shown in Fig. 1.

### Computed tomography protocol

All MDCT examinations of the chest before surgery were performed using the Discovery CT750 HD scanner (General Electrical Medical Systems, Milwaukee, WI, USA). Generally, the scan began at 2.0 cm above the lung apices and extended through the adrenal glands. The following imaging parameters were used: 120 kVp tube voltage; autoregulation of mA (200–400 mA) and noise index of 9; detector collimation of 64 × 1.25 mm; 0.6 s/ rotation gantry rotation speed; and helical pitch of 0.984. Axial images were reconstructed using a section width of 5.0 mm. Coronal and sagittal reformations were reconstructed using a section width of 5.0 mm. After the plain scan, the non-ionic contrast medium Iohexol (Omnipaque 300; GE Healthcare) at a dose of 1.5 mL/kg body was injected at a rate of 3.0 mL/s through the median cubital vein. The enhanced scan was conducted 30 s (arterial phase) and 55 s (delayed phase) after the start of the contrast medium injection.

### Image analysis

The CT images were reviewed by two radiologists (Dr. Yan and Dr. Wei with 5 years and 8 years of experience in thoracic CT, respectively). The two reviewers were unaware of the final pathologic information of the patient. They assessed the qualitative characteristics of each lesion on the plain scan, arterial and delayed phases with consensus. Any discrepancy during analysis was resolved through achieving consensus by consulting a senior thoracic radiologist (Dr. Shi, 12 years of experience reading thoracic CT). Quantitative variables were recorded as the average of two separate measurements by two radiologists (Dr. Yan and Dr. Wei).

### Qualitative analysis

**Fig. 2:**
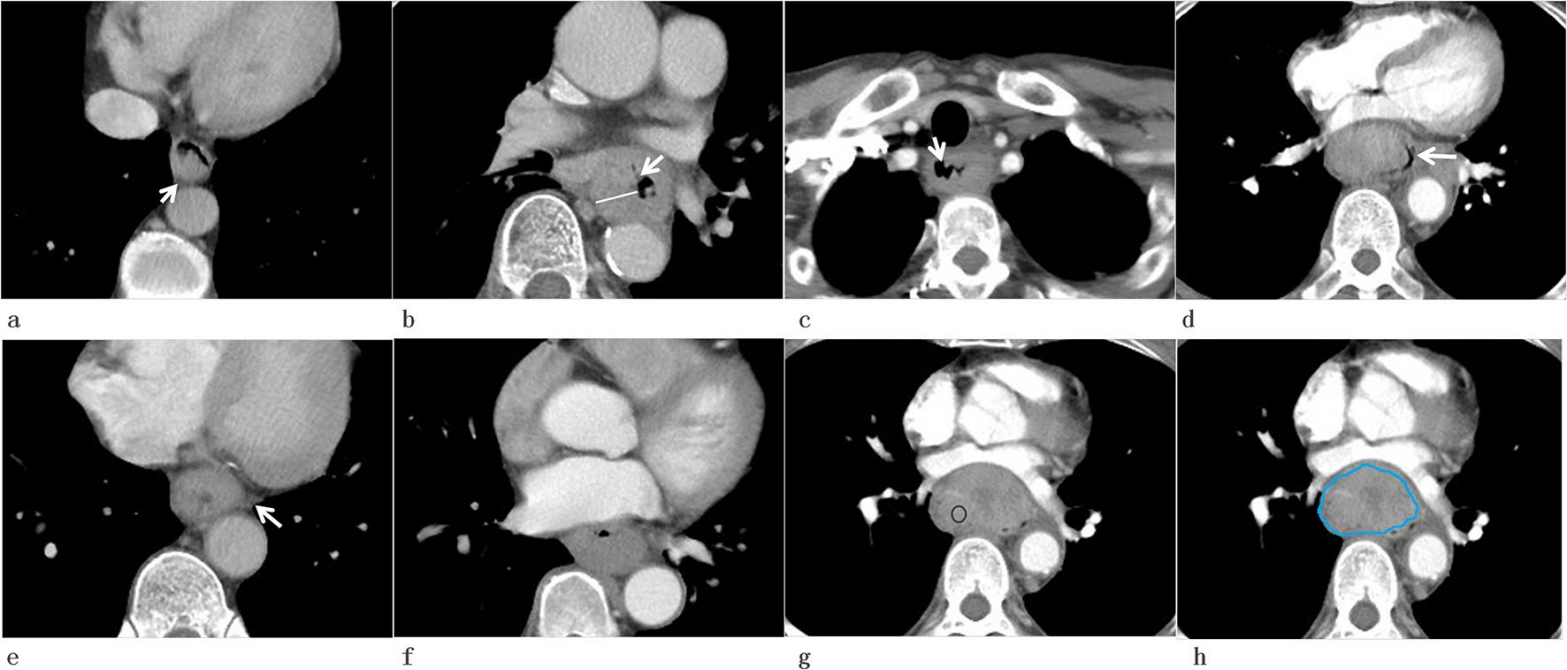
CT signs of esophageal tumor. **a** One focal esophageal lesion with tumor range of 0–1/2 (arrow). **b** One diffuse type lesion with tumor range of 1/2–1 with deep ulcer in anterior wall (arrow), the white line showed the measurement of thickness of tumor. **c** One diffuse growth lesion with non-smooth tumor air surface (arrow). **d** One expansive esophageal lesion with smooth tumor air surface (arrow). **e** One esophageal lesion with fibrosis of peritumoral fat space (arrow). **f** One esophageal lesion with homogeneous intensity. **g**, **h** One esophageal lesion with heterogeneous intensity with ROI of maximum enhanced intensity (**g**) and ROI of encompassing the tumor on the maximal section (**h**)

The following qualitative CT findings were analyzed: (1) location (neck, upper-thorax, middle-thorax, and low-thorax of the esophagus); (2) tumor range (0–1/2 and 1/2–1), for some focal lesion, double-contrast barium esophagogram was performed for assessing the tumor range; If the tumor occupied 0 to a half of the esophageal wall, the tumor range would be defined as type of 0–1/2; then, if the tumor occupied a half to whole of the esophageal wall, the tumor range would be defined as type of 1/2–1; (3) enhancement pattern (homogeneity and heterogeneity); the tumor appearing homogeneous enhancement was defined as homogeneity of enhancement pattern and the tumor appearing heterogeneous enhancement was defined as heterogeneity of enhancement pattern in arterial and delayed phases; (4) tumor air surface (smooth, or non-smooth); (5) necrosis in tumor (absence, or presence); non-enhanced area in the tumor was defined as necrosis; (6) fibrosis of peritumoral fat space (absence, or presence); increased focal stranding in periesophageal fat, which showed esophageal infiltration of tumor, was defined as fibrosis of peritumoral fat space; (7) ulcer of tumor (absence, or presence) was diagnosed by the presence of air in the esophageal wall or focal necrosis from lumen to the deep level of the wall, not perforated the wall (Fig. 2). A diameter of lymph node larger than 5 mm, or the presence of necrosis in LNs, or irregular margin of LNs, or LNs near the tumor was diagnosed as metastatic LN. Number of metastatic LN was also recorded.

### Quantitative analysis

Wall thickness of esophageal tumor was measured perpendicular to the lumen on the axial images in delayed phase. If the lumen was not visible, the maximal tumor diameter was obtained and multiplied by 0.5. Tumor length (the tumor’s longest diameter, L-tumor) was measured at sagittal CT imaging in delayed phase. The diameters of the short (SD-LLN) and long axis (LD-LLN) of largest lymph node were measured on the axial images in delayed phase. The regions of interest (ROIs) were manually drawn to encompass the tumor on the maximal section in axial images in delayed phase and copied the ROIs on the same slice in the other two phases. The type of ROIs was irregular. Area of tumor in largest slice (Area _max-axial_) and mean CT value of maximum axial area of tumor were obtained through ROIs of axial images. The mean CT attenuation values of tumor were recorded as the N CT value mean, AP CT value mean, and DP CT value mean, respectively. The postcontrast attenuation differences of tumor (ΔAP-N CT value mean, ΔDP-N CT value mean, and ΔDP-AP CT value mean) were calculated. The ROIs were drawn to encompass the area of greatest enhancement on the maximal section in the arterial or delayed phase and copied the ROIs on the same slice in the other two phases. The type of these ROIs was circular. The maximum CT values of tumor were obtained through ROIs of axial images. The maximum CT attenuation values of tumor were recorded as the N CT value max, AP CT value max, and DP value max, respectively. The postcontrast attenuation differences of tumor (ΔAP-N CT value max, ΔDP-N CT value max, and ΔDP-AP CT value max) were calculated.

### Pathological evaluation

After surgery or biopsy, all esophageal specimens were processed according to standard pathological procedures. The histopathologic diagnosis of esophageal tumor was determined using microscopy by an experienced pathologist (Dr. Yang). The results of the analysis of the surgical specimen served as a standard of reference.

### Statistical analysis

Differences in quantitative parameters in patients with PMME, SCC and esophgeal leiomyoma were assessed by the independent *t* test or Mann–Whitney test according to the condition of normal distribution. Categorical parameters were compared between PMME, SCC and leiomyoma by Chi-square test or Fisher’s exact test. Multivariate logistic regression was performed to select independent factors for discrimination between PMME, SCC and leiomyoma and to establish a predictive models. Receiver operating characteristic (ROC) curve analysis was applied to evaluate the diagnostic performance for differentiating PMME from leiomyoma and SCC, with the area under the ROC curve (AUC) calculated as well as sensitivity, specificity, positive predictive value (PPV), negative predictive value (NPV), and accuracy. Nomogram was constructed. Calibration curves were yielded with Hosmer–Lemeshow test. Intraclass correlation coefficients (ICCs) were determined to evaluate inter-observer agreement in terms of parameter extraction. A coefficient of 0.81–1.00 indicated an almost perfect agreement; 0.61–0.80, 0.41–0.60, 0.21–0.40, and 0–0.2 reflected substantial, moderate, fair, and poor/no agreement, respectively. Data analysis was conducted with SPSS 22.0 (IBM Corporation, Armonk, NY, USA) and R package 3.6.2 (R Foundation for Statistical Computing, Vienna, Austria). A two-sided *P* < 0.05 indicated statistical significance.

## Results

### Characteristics of the patients

Among 23 patients with PMME, 13 patients underwent thoracotomy with tumor enucleation. Other patients with biopsy-proven PMME were not operative candidates because of the health issues or unresectable tumors. The clinical characteristics of the patients are summarized in Table 1. Two radiologists independently delineated the ROIs of the esophageal tumors and achieved satisfactory agreement. Quantitative CT analyses between the two radiologists showed perfect or substantial agreement with ICCs of 0.75–0.89.

**Table 1:** Univariate analysis of demographic data and CT findings among the PMME, SCC, and leiomyoma

Characteristics	PMME		SCC	Leiomyoma	*P* (PMME vs SCC)	*P* (PMME vs Leiomyoma)
Clinical characteristics						
Age (years)	57.09 ± 11.50		62.45 ± 7.08	45.95 ± 10.49	0.086	0.004
Sex, *n* (%)					0.004	0.053
Male	10 (43.5)		54 (78.3)	16 (76.2)		
Female	13 (56.5)		15 (21.7)	5 (23.8)		
Qualitative analysis						
Location, *n* (%)					0.238	0.800
Neck	0 (0)		3 (4.4)	0 (0)		
Upper-thorax	4 (17.4)		4 (5.8)	6 (28.6)		
Mid-thorax	7 (30.4)		33 (47.8)	4 (19.0)		
Low-thorax	12 (52.2)		29 (42)	11 (52.4)		
Tumor range, *n* (%)					0.002	0.255
0–1/2	8 (34.8)		3 (4.3)	12 (57.1)		
1/2–1	15 (65.2)		66 (95.7)	9 (42.9)		
Enhancement pattern, *n* (%)					0.036	0.020
Homogeneous	14 (60.9)		59 (85.5)	20 (95.2)		
Heterogeneous	9 (39.1)		10 (14.5)	1 (4.8)		
Tumor air surface, *n* (%)					0.793	0.045
Smooth	14 (60.9)		37 (53.6)	19 (90.5)		
Non-smooth	9 (39.1)		32 (46.4)	2 (9.5)		
Necrosis of tumor, *n* (%)					0.020	0.008
Absence	15 (65.2)		62 (89.9)	20 (95.2)		
Presence	8 (34.8)		7 (10.1)	1 (4.8)		
Fibrosis of peritumoral fat space, *n* (%)					0.006	0.847
Absence	20 (87.0)		36 (52.2)	20 (95.2)		
Presence	3 (13.0)		33 (47.8)	1 (4.8)		
Ulcer, *n* (%)					0.749	0.911
Absence	18 (78. 3)		49 (71.0)	18 (85.7)		
Presence	5 (21.7)		20 (29.0)	3 (14.3)		

*n* number, *PMME* primary malignant melanoma of esophagus, *SCC* squamous cell carcinoma

### Univariable comparisons of qualitative and quantitative analysis

Tables 1 and 2 show the univariable comparisons of qualitative and quantitative analysis for differentiating PMME from SCC and leiomyoma. The qualitative analysis showed that tumor range, enhancement pattern, necrosis of tumor, and fibrosis of peritumoral fat surface were significantly different between PMME and SCC. Tumor range of 0–1/2, heterogeneous enhanced pattern, necrosis of tumor, and the absence of fibrosis of peritumoral fat space were often observed in PMME group compared with SCC (Table 1). In CT quantitative analysis, there were significant differences in thickness of tumor, AP CT value mean and max, and so on between PMME and SCC (Table 2). The qualitative analysis showed that enhanced pattern, tumor air surface and necrosis of tumor were statistical different between PMME and esophageal leiomyoma. Heterogeneous enhanced pattern, necrosis of tumor and non-smooth tumor surface were often observed in PMME group compared with esophageal leiomyoma. In CT quantitative analysis, there were significant differences in short axis diameter of largest lymph node, number of metastatic lymph node, the mean and maximal CT value of tumor in delayed phase, and ΔAP-N CT value max between PMME and leiomyoma (*P* < 0.001) (Table 2).

**Table 2:** Univariate analysis of quantitative CT parameters among the PMME, SCC, and leiomyoma

Measurements	PMME	SCC	Leiomyoma	*P* (PMME vs SCC)	*P* (PMME vs Leiomyoma)
THK-tumor (mm)	22.13 ± 9.20	14.20 ± 4.09	30.33 ± 26.70	< 0.001	0.660
L-tumor (mm)	52.78 ± 27.99	53.51 ± 21.29	39.05 ± 25.03	0.989	0.181
LD-LLN (mm)	18.48 ± 9.32	15.84 ± 5.45	11.62 ± 4.07	0.647	0.012
SD-LLN (mm)	11.91 ± 8.12	8.96 ± 3.86	4.71 ± 1.27	0.483	< 0.001
Number of MLN (n)	1.26 ± 1.42	1.39 ± 1.51	0.00 ± 0.00	0.958	< 0.001
Area of tumor (cm^2^)	645.87 ± 513.03	399.07 ± 206.78	647.67 ± 1354.10	0.179	0.238
N CT V mean(HU)	35.61 ± 10.71	25.94 ± 12.11	33.21 ± 16.94	0.004	> 0.999
AP CT V mean(HU)	61.87 ± 19.53	47.65 ± 15.42	45.80 ± 15.87	0.004	0.042
DP CT V mean(HU)	62.61 ± 14.03	59.18 ± 15.34	45.33 ± 15.93	0.793	< 0.001
ΔAP-N CT V mean (HU)	26.26 ± 19.03	21.71 ± 13.48	10.57 ± 21.70	0.777	0.004
ΔDP-N CT V mean (HU)	27.00 ± 8.87	33.24 ± 14.52	16.71 ± 16.96	0.047	0.016
ΔDP-AP CT V mean(HU)	0.74 ± 14.87	11.53 ± 14.73	6.14 ± 31.65	0.010	0.535
N CT V max (HU)	40.83 ± 11.65	33.04 ± 11.65	41.14 ± 11.13	0.010	0.877
AP CT V max (HU)	75.22 ± 16.50	58.06 ± 18.59	51.50 ± 18.45	0.005	0.006
DP CT V max (HU)	72.87 ± 14.62	67.90 ± 16.02	55.05 ± 14.73	0.172	< 0.001
ΔAP-N CT V max (HU)	34.39 ± 15.85	25.01 ± 18.33	11.00 ± 5.20	0.026	< 0.001
ΔDP-N CT V max (HU)	32.04 ± 12.22	34.85 ± 14.99	17.62 ± 15.06	0.669	0.321
ΔDP-AP CT V max (HU)	− 2.35 ± 13.79	9.84 ± 20.08	5.62 ± 4.50	0.002	0.006

*AP* arterial phase, *CT V* computed tomography value, *DdP* delayed phase, *HU* Hounsfield Unit, *LD-LLN* long diameter of the largest lymph node, *L-tumor* length of tumor, *max* maximum, *MLN* metastatic lymph node, *n* number, *N* non-enhancement, *PMME* primary malignant melanoma of esophagus, *SD-LLN* short diameter of the largest lymph node, *SCC* squamous cell carcinoma, *THK-tumor* thickness of tumor, Δ postcontrast attenuation difference of tumor

### Diagnostic performance of CT model for differentiating PMME and SCC

**Fig. 3:**
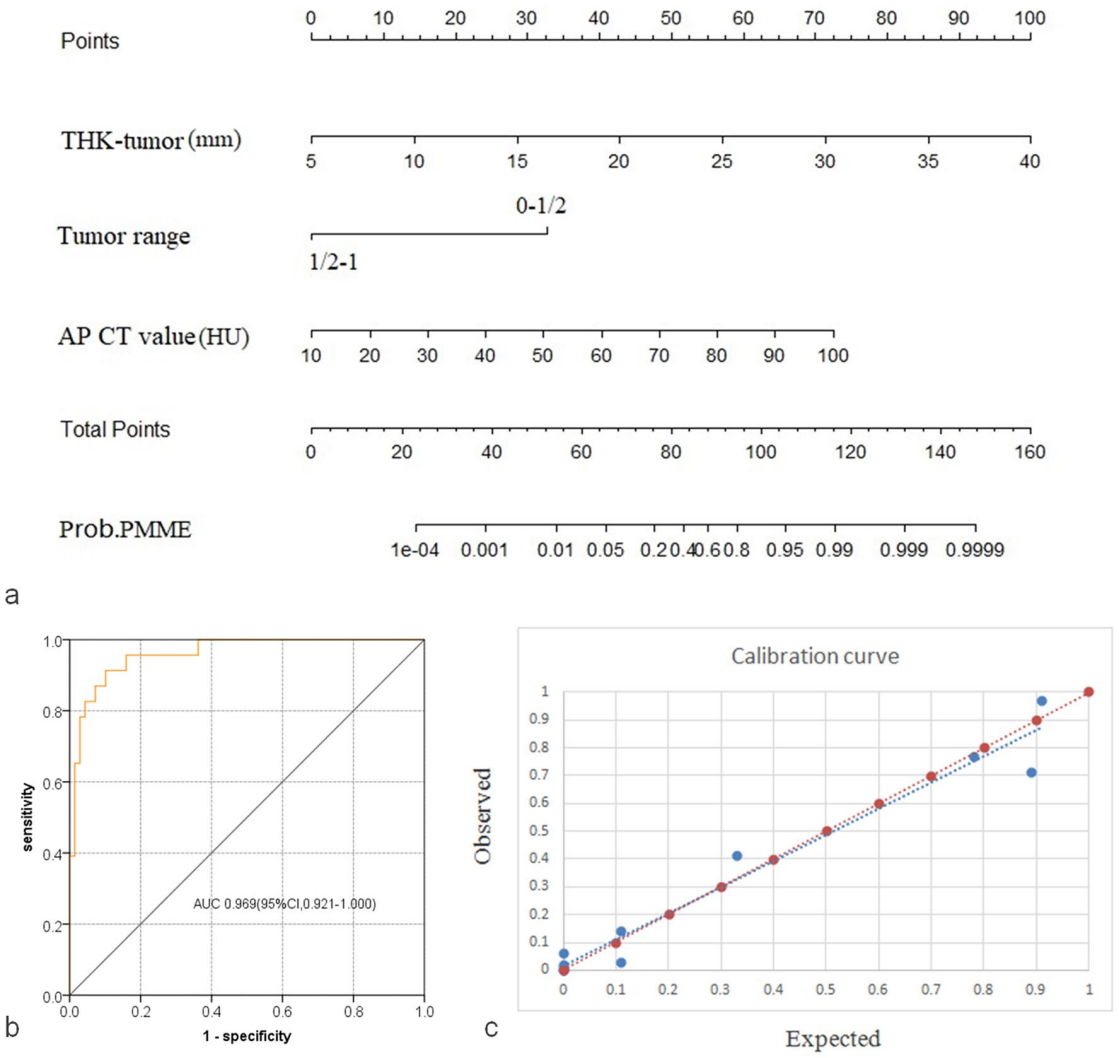
Nomogram based on multivariate models for differentiating PMME from SCC using CT findings, receiver operating characteristics curve, and the corresponding calibration curves. **a** The developed nomogram. **b** The AUC of nomogram for discriminating PMME from SCC was 0.969. **c** Calibration curves depicted the calibration of nomogram in terms of the agreement between the predicted probability of PMME and actual outcomes of the PMME. The y axis represented the actual probability of PMME. The *x* axis represented the predicted probability of PMME. The red line represented a perfect prediction by an ideal model. The blue line showed the performance of the CT model. The blue line was closer to the red line, which suggested a better prediction

Table 3 shows the adjusted logistic regression models for differentiating PMME from SCC and leiomyoma. We found that the thickness of the tumor, AP CT value mean, and tumor range were independent factors for differentiating PMME from SCC. This analysis revealed that for differentiation between PMME and SCC, the thickness of the tumor had a higher rate of diagnosing PMME (Table 3). The performances of thickness of tumor, AP CT value mean, and tumor range for differentiating PMME from SCC are shown in Table 4. So, thickness of esophageal tumor, AP CT value mean, and tumor range were used to establish a diagnostic model. A combined CT model was established using the following formula: value = 0.423 × thickness of esophageal tumor + 0.119 × AP CT value mean − 4.857 × tumor range. Regarding to tumor range, 0–1/2 was assigned as 1 and 1/2–1 was assigned as 2. The combined CT diagnostic model producing value larger than the cufoff value of 5.7 for diagnosing PMME yielded AUC of 0.960 (Fig. 3).

**Table 3:** Multivariable logistic regression results of CT parameters for differentiating PMME from SCC

Parameters	B	OR	95% CI	*P*
THK-tumor (mm)	0.423	1.527	1.234–1.889	< 0.001
AP CT value mean ( HU)	0.119	1.127	1.043–1.217	0.002
Tumor range	− 4.857	0.008	0.001–0.114	< 0.001

*B* regression coefficient, *CI* confidence interval, *HU* Hounsfield Unit, *OR* odds ratio, *THK-tumor* thickness of tumor

**Table 4:** Performance of CT model for differentiating PMME from SCC and leiomyoma

	AUC	Cutoff	SEN	SPE	PPV	NPV	ACU
*PMME vs SCC*							
CT model	0.969	5.7*	87.0%	91.3%	76.9%	95.5%	90.2%
	(0.921–1.000)		(20/23)	(63/69)	(20/26)	(63/66)	(83/92)
THK-tumor	0.750	18 mm*	65.2%	81.2%	58.1%	91.8%	80.4%
	(0.614–0.887)		(15/23)	(56/69)	(18/31)	(56/61)	(74/92)
AP CT value mean	0.710	56HU*	60.9%	75.4%	45.2%	85.2%	71.7%
	(0.586–0.835)		(14/23)	(52/69)	(14/31)	(52/61)	(66/92)
Tumor range	0.652	(0–1/2)**	34.8%	95.7%	72.7%	81.5%	80.4%
	(0.509–0.795)		(8/23)	(66/69)	(8/11)	(66/81)	(74/92)
*PMME vs leiomyoma*							
ΔAP-N CT value max	0.929	16HU*	82.6%	90.5%	90.5%	82.6%	86.4%
	(0.851–1.100)		(19/23)	(19/21)	(19/21)	(19/23)	(38/44)

*ACU* accuracy, *AP* arterial phase, *AUC* area under curve, *CT* computed tomography, *NPV* negative predictive value, *PMME* primary malignant melanoma of esophagus, *PPV* positive predictive value, *SCC* squamous cell carcinoma, *SEN* Sensitivity, *SPE* Specificity, *THK-tumor* thickness of tumor, ΔAP-N CT value max, postcontrast attenuation difference between AP and non-enhancement scan

*The value larger than cutoff value indicated the diagnosis of PMME

**The tumor range with 0–1/2 indicated the diagnosis of PMME

The multivariable analysis showed that ΔAP-N CT value max was an independent factor for differentiating PMME from esophageal leiomyoma. The performance of this parameter larger than cutoff value of 16HU for diagnosing PMME from leiomyoma was well with AUC of 0.929. Detailed information on the performance of the combined CT models for distinguishing PMME from SCC and leiomyoma is shown in Table 4.

### Clinical usefulness

To provide clinicians with an easy tool, a nomogram based on quantitative and qualitative CT parameters, including thickness of tumor, range of tumor, and CT mean value of AP, was developed (Fig. 3). The calibration curve of CT model estimating the probability of PMME demonstrated excellent agreement. Another nomogram based on ΔAP-N CT value max was also developed and the calibration curve of this model estimating the probability of PMME demonstrated excellent agreement (Fig. S1). The probability of differentiating PMME from SCC or esophageal leiomyoma ranged from 0 to 1. A probability nearing 1 indicated high odds of PMME. Patients with esophageal tumor could benefit from these diagnosing models.

### Comparison of pathology between PMME, SCC, and esophageal leiomyoma

**Fig. 4:**
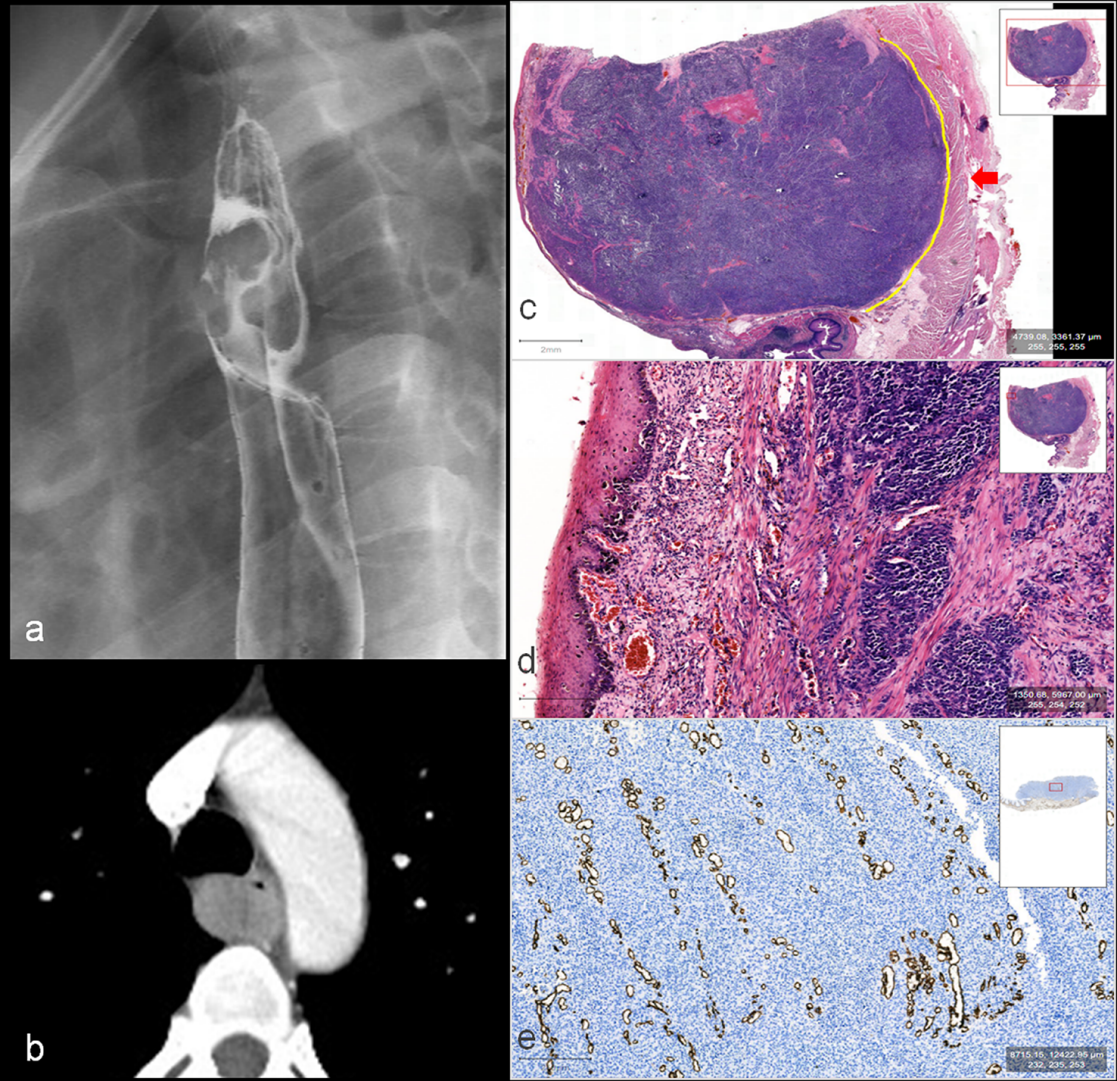
53-year-old woman with PMME. **a** Right posterior oblique spot image from double-contrast barium esophagogram showed a smooth submucosal mass of posterior wall with tumor range of 0–2/1 in upper thoracic esophagus compressing lumen without obstruction. **b** Arterial CT showed a rounded well-circumscribed enhanced mass with mean CT value of 87HU and thickness of 19 mm; according to the CT model, the value was 13.53 indicating the diagnosis of PMME (larger than the cutoff value of 5.7); according to nomogram, total points of this esophageal tumor (19 mm corresponding to 40 points, 87 HU corresponding to 60 point, and tumor range of 0–1/2 corresponding to 33points) were 133 and the probability of diagnosing PMME was 0.999. **c** The hematoxylin and eosin (H&E) staining showed the tumor with expansive growth pattern and clear boundary (yellow solid line) and remaining intact muscle layer (red arrow). **d** It showed the intact surface squamous epithelium and melanocytes located at the base of squamous epithelium. **e** CD34 immunohistochemical staining showed that there were abundant small blood vessels in the sheet of tumor cells

**Fig. 5:**
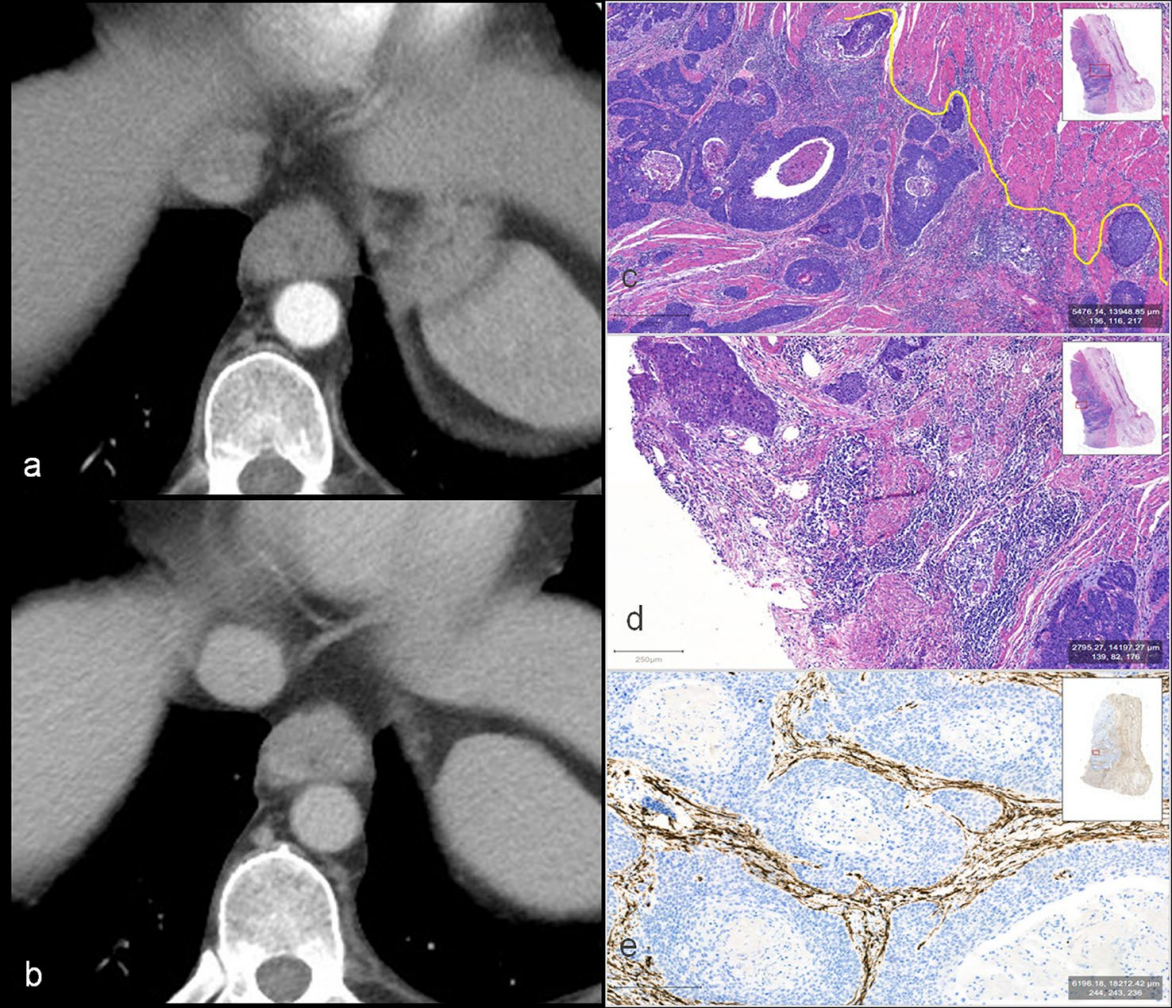
71-year-old man with SCC. **a** Arterial CT showed a slightly enhanced and infiltrative tumor with mean CT value of 50 HU and thickness of 15 mm. **b** It showed progressive enhancement with 86 HU in delayed phase; according to the CT model, the value was 2.58 indicating the diagnosis of SCC (less than the cutoff value of 5.7); according to nomogram, total points of this esophageal tumor (15 mm corresponding to 28 points, 50 HU corresponding to 34 point, and tumor range of 1/2–1 corresponding to 0 point) were 62 and the probability of diagnosing PMME was 0.04 indicating the diagnosis of SCC. **c** The hematoxylin and eosin (H&E) staining showed the tumor with infiltrative growth in the esophageal wall (the yellow solid line represents the invasive boundary), and the tumor cell nest was surrounded by connective tissue stroma with abundant fiber and lymphoid cells. **d** It showed that no squamous epithelium remained on the surface of the tumor. **e** CD34 immunohistochemical staining showed that small blood vessels were not detected in tumor nests but concentrated in the stroma surrounded them

The PMME arose from the melanocytes in the basal layer of squamous epithelium. Tumor cell nests could be seen in the basal layer of squamous epithelium at the junction of tumor and normal esophagus, suggesting that the tumor originated from esophagus. Regarding its gross appearance, PMME often presented a broad base polypoid appearance which often grew expansively in submucosa layer and rarely eroded the entire mucosal epithelium (Fig. 4). The surface mucosa often formed focal erosion or superficial ulcer due to expansion tension and mechanical friction with food. However, SCC was a tumor arising from squamous epithelium usually involving the whole layer of squamous epithelium and infiltrating to the deep layer, therefore with generalized mural thickening with a real ulcer (Fig. 5).

Microscopically, PMME was mainly composed of sheet of melanoma cells with rare stroma reaction. The boundary of PMME between tumor area and peripheral normal tissue was usually smooth and clear with involving a small range of esophageal wall (Fig. 4). The infiltration of SCC was accompanied by promoting stromal fibrosis connective tissue proliferation, and characterized by abundant stroma around the tumor nests. SCC commonly showed diffuse infiltration with an infiltrative boundary, involving a wide range, mostly the entire layers of the esophageal wall in the current cohort (Fig. 5).

Focal necrosis was a common morphological feature in both PMME and SCC. For blood supply, small vessels were easy to be observed in tumor sheet of PMME, but only presented in the fibrous stroma around the tumor nests for SCC. Interstitial vessels in PMME were more abundant than that of SCC in CD34 immunohistochemical staining (Figs. 4, 5). Regarding to immunohistochemistry of PMME, most of the tumor cells were HMB45 positive, Melan A positive, S100 positive, and CK negative, and the number of Ki67 positive cells ranged between 40 and 50% [18].

**Fig. 6:**
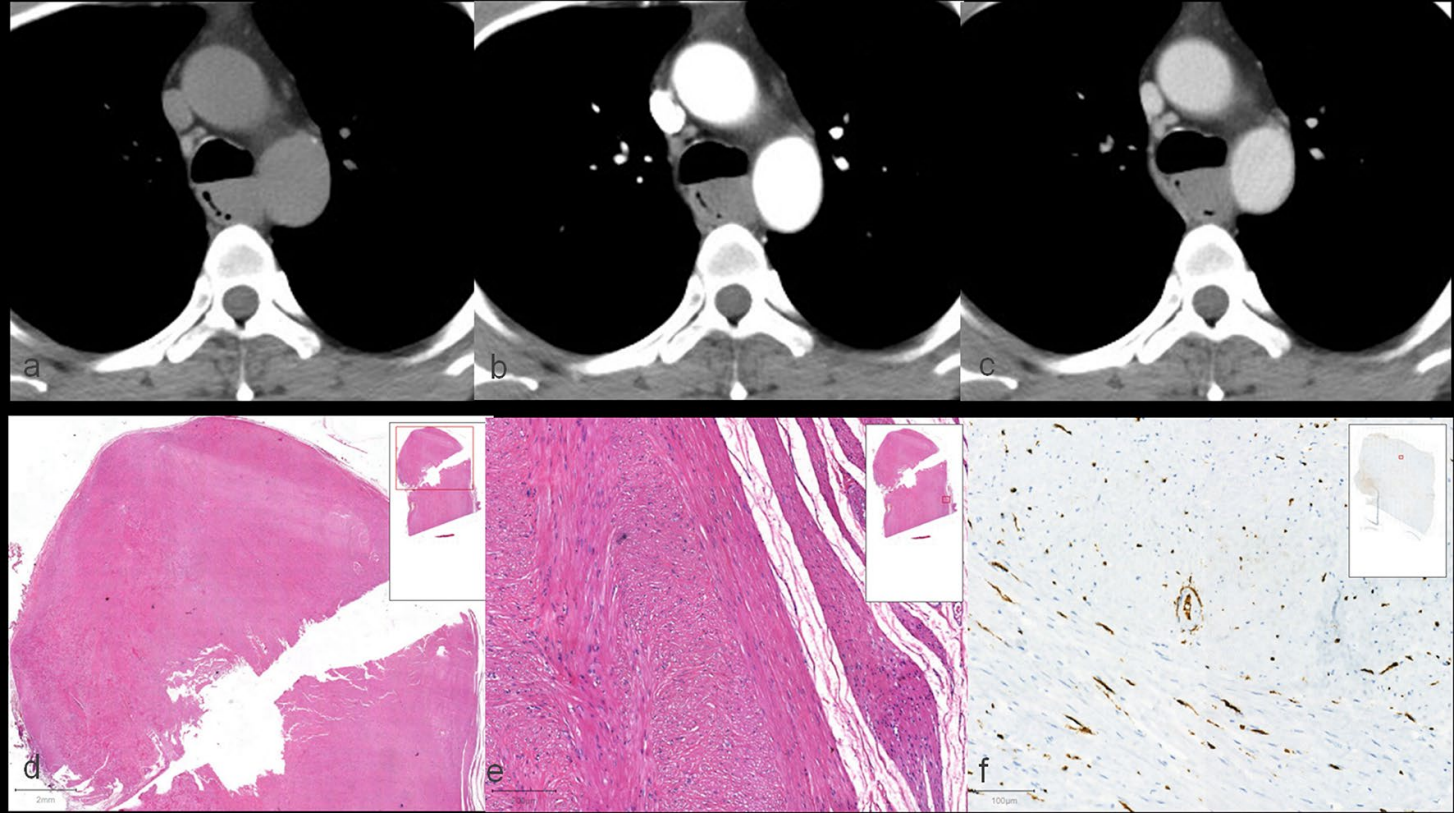
43-year-old man with esophageal leiomyoma. **a** Plain CT showed a rounded well-circumscribed homogeneous mass with maximal CT value of 51HU. **b** Arterial CT showed a slightly enhanced tumor with maximal CT value of 57 HU. **c** It also showed slightly enhancement with 54 HU in delayed phase. ΔAP-N CT value max was 6 HU indicating the diagnosis of leiomyoma (less than the cut off value of 16 HU). **d** The hematoxylin and eosin (H&E) staining showed the well-defined masses in low magnification imaging. **e** It showed that tumors with smooth muscle cells that originated from the muscularis propria in high magnification imaging. **f** CD34 immunohistochemical staining showed that the leiomyoma outlined sparse blood vessels in the tumor

Grossly, leiomyoma appeared as a well-defined mass in the esophageal wall and had a solid, grayish white appearance on cross section. Ulceration of the overlying mucosa was uncommon. Microscopically, leiomyoma had the usual characteristics of a benign smooth muscle tumor with a clear boundary and stain for muscle markers such as desmin and smooth muscle actin. Local resection or enucleation was usually successful. Blood vessels of the leiomyoma were fewer than that of squamous cell carcinoma and melanoma (Fig. 6).

## Discussion

The treatment of choice for PMME was surgical resection with dissection of the lymph nodes [19]. Because of the propensity for submucosal spread of PMME, the surgical procedure should include a radical procedure with a greater margin than that used for the usual SCC [16], and in PMME, aggressive lymph node dissection was beneficial for accurate staging, potentially reducing recurrence and improving survival [20, 21]. So, it was important to obtain a preoperative diagnosis of PMME for choosing more aggressive surgery. This study found that thickness of esophageal tumor, AP CT value mean, and tumor range showed significant difference between PMME and SCC. This diagnosing model yielded perfect performance for distinguishing PMME and SCC. To the best of our knowledge, few studies focused on CT analysis and pathologic comparison for diagnosing PMME. To date, the present study was the largest population of PMME patients regarding radiological finding with more than 10 years span.

In this study, we found that PMME tended to appear intraluminal expansively growth pattern, which meant a broad base polypoid appearance mass protruding to the lumen with well-circumscribed boundary and smooth surface. In contrast, diffuse thickening and infiltrative growth pattern were more likely to occur in SCC lesions. It was consistent with other reports [16, 17]. Previous report found that in PMME, ulcerations may be present, but more often the tumor was covered with intact mucosa [16]. We also found that there was no difference in the presence of ulceration between PMME and SCC. The superficial ulcer was commonly observed in PMME, while deep ulcer due to tumor infiltrating to the esophageal wall was often presented in SCC. Small focal necrosis was a common manifestation in both PMME and SCC, which could not be used as the distinguishing point for this differentiation.

Our study showed that the thickness of PMME was higher than that of SCC and was one of the independent parameters for diagnosing PMME. The expansive growth type may result in the high size in PMME. The origination from basal layer, and expansive growth type in PMME may contribute to less esophageal symptoms and result in the higher size at initial diagnosis. Significant differences in CT value-related parameters were AP CT value mean and max between PMME and SCC in univariable analysis. We also found that PMME more often presented obvious enhancement in arterial phase, and SCC was prone to show progressive enhancement and enhanced peak in delayed phase. Tang et al. also found that enhanced CT scanning revealed obvious lesion enhancement, suggesting a rich tumor blood supply [18]. The other previous reports also found that contrast-enhanced CT showed malignant melanoma uneven enhanced in liver and stomach [22, 23]. In our study, interstitial vessels in PMME were more abundant than that of SCC in CD34 immunohistochemical staining. So, more interstitial vessels in PMME contributed to obvious enhancement in arterial phase. Rich fibrosis component in SCC was associated with peak enhancement in delayed phase.

Leiomyoma was the most common benign tumors of the esophagus [24]. The majority of esophageal leiomyoma originated from the inner circular muscle and two-thirds of this tumor was located in the middle or low thoracic esophagus [8]. Sometimes, PMME may be misdiagnosed as esophageal leiomyoma. In this study, we found that compared with PMME, esophageal leiomyoma often showed a smoothly marginated homogeneous mass with slightly homogeneous enhancement in arterial and delayed phases. Metastatic lymph nodes were not observed in esophageal leiomyoma. These results in our study were consistent with that of previous studies [8, 25]. In addition, ΔAP-N CT value max of tumor was an independent factor for differentiating PMME from esophageal leiomyoma. The AUC of ΔAP-N CT value max larger than the cutoff value of 16HU for diagnosing PMME from leiomyoma was 0.929. The robustness of our study was the use of quantitative parameter for this differentiation with high efficiency.

Even if the endoscopic biopsy was used, the preoperative diagnostic accuracy of PMME was only about 80% [4]. Sometimes the histological diagnosis of PMME can be challenging for several reasons. First, approximately 10–25% of PMME cases presented various colors, such as purple, brown, and white, depending on the melanin quality [5]. Second, when biopsy specimen lacked of melanin granules, the tumor might be incorrectly diagnosed as an epithelial carcinoma. Third, because of PMME arising from the melanocytes in the basal layer of squamous epithelium, the superficial biopsy may produce negative result. This study showed that morphological characteristics combined with CT value parameters were effective in diagnosing PMME.

In terms of clinical use, the radiologists could consider the result of diagnostic models in diagnosing PMME according to the probability of PMME from 0 to 1. The parameters of an esophageal tumor were applied to the two models, respectively. If there was a difference in diagnosis of PMME between the two models, the subjective diagnosis was needed to correct the diagnosis by combining radiological findings and clinical features. The differentiation between SCC and esophageal leiomyoma was evaluated by radiologists, considering the clinical and radiological features. When esophageal tumors were difficult to diagnose by radiologists assisted by our models, we recommend further evaluation with endoscopic ultrasound (EUS) and fine-needle aspiration (FNA) to look for the presence of PMME. When our CT analysis suggested a diagnosis of PMME for the esophageal lesion, but the histological diagnosis cannot identified in the initial biopsy, the immunohistochemical analysis of biopsy specimen or a deeper esophageal biopsy should be performed to obtain an accurate diagnosis.

There were several limitations in this study. First, it was a single-center retrospective study, and sample ratio might be biased. Second, because of the rarity, our study only included a small number of patients of PMME; a much larger database from multicenter with considerably larger sample was needed to validate the robustness and reproducibility of our result. Third, not all patients received surgery and the resected specimens were not allowed for pathological analysis because the PMME had aggressive biological behavior and these patients without receiving surgery were diagnosed late. Fourth, among these non-epithelial neoplasms, esophageal leiomyoma was the most common mesenchymal tumor of esophagus, unlike in the gastrointestinal tract, where GISTs predominated; the other non-epithelial neoplasms were extremely rare [8]. So, we only chose esophageal leiomyoma as a control group to differentiate from PMME. Searching for the radiologic finding of the other non-epithelial neoplasms is another study.

Comparing with SCC, PMME was prone to manifest expansive growth pattern, more arterial enhancement, larger tumor thickness, and less infiltrative range. This CT model might hold promise in discriminating PMME from SCC. Comparing with leiomyoma, PMME showed more obvious heterogeneous enhancement pattern, the presence of malignant lymph nodes, necrosis in the tumor, and non-smooth surface of tumor. These CT models might hold promise in discriminating PMME from SCC and esophageal leiomyoma. Although PMME was less common than SCC, radiologists should be familiar with the imaging and pathologic features of PMME as well as their malignant behavior and appropriate patient management.

## Supplementary Information

Below is the link to the electronic supplementary material.

Supplementary file1 (TIF 1710 KB) Fig. S1 Nomogram based on ΔAP-N CT value max for differentiating PMME from esophageal leiomyoma and the corresponding calibration curves. (a) The developed nomogram. (b) Calibration curves depicted the calibration of nomogram in terms of the agreement between the predicted probability of PMME and actual outcomes of the PMME. The y axis represented the actual probability of PMME. The x axis represented the predicted probability of PMME. The red line represented a perfect prediction by an ideal model. The blue line showed the performance of the CT model. The blue line was closer to the red line, which suggested a better prediction.

## Abbreviations

AP: Arterial phase
DP: Delayed phase
LD-LLN: Long diameter of the largest lymph node
L-tumor: Length of tumor
MLN: Metastatic lymph node
PMME: Primary malignant melanoma of the esophagus
SD-LLN: Short diameter of the largest lymph node
SCC: Squamous cell carcinoma
THK-tumor: Thickness of tumor

## References

[1] Volpin E, Sauvanet A, Couvelard A, Belghiti J (2002). Primary malignant melanoma of the esophagus: a case report and review of the literature. Dis Esophagus.

[2] Kido T, Morishima H, Nakahara M, Nakao K, Tanimura H, Nishimura R, Tsujimoto M (2000). Early stage primary malignant melanoma of the esophagus. Gastrointest Endosc.

[3] Iwanuma Y, Tomita N, Amano T, Isayama F, Tsurumaru M, Hayashi T, Kajiyama Y (2012). Current status of primary malignant melanoma of the esophagus: clinical features, pathology, management and prognosis. J Gastroenterol.

[4] Chen H, Fu Q, Sun K (2020). Characteristics and prognosis of primary malignant melanoma of the esophagus. Medicine (Baltimore).

[5] Wang X, Kong Y, Chi Z, Sheng X, Cui C, Mao L, Lian B, Tang B, Yan X, Si L, Guo J (2019). Primary malignant melanoma of the esophagus: a retrospective analysis of clinical features, management, and survival of 76 patients. Thorac Cancer.

[6] Liu H, Yan Y, Jiang CM (2016). Primary malignant melanoma of the esophagus with unusual endoscopic findings a case report and literature review. Medicine (Baltimore).

[7] Hashimoto T, Makino T, Yamasaki M, Tanaka K, Miyazaki Y, Takahashi T, Kurokawa Y, Motoori M, Kimura Y, Nakajima K, Morii E, Mori M, Doki Y (2019). Clinicopathological characteristics and survival of primary malignant melanoma of the esophagus. Oncol Lett.

[8] Lewis RB, Mehrotra AK, Rodriguez P, Levine MS (2013). From the radiologic pathology archives: esophageal neoplasms: radiologic-pathologic correlation. Radiographics.

[9] Mujawar P, Pawar T, Chavan RN (2016). Video assisted thoracoscopic surgical enucleation of a giant esophageal leiomyoma presenting with persistent cough. Case Rep Surg.

[10] Beji H, Bouassida M, Kallel Y, Tormane MA, Mighri MM, Touinsi H (2022). Leiomyoma of the esophagus: a case report and review of the literature. Int J Surg Case Rep.

[11] Konieczny A, Meyer P, Schnider A, Komminoth P, Schmid M, Lombriser N, Weishaupt D (2013). Accuracy of multidetector-row CT for restaging after neoadjuvant treatment in patients with oesophageal cancer. Eur Radiol.

[12] Cerfolio RJ, Bryant AS, Ohja B, Bartolucci AA, Eloubeidi MA (2005). The accuracy of endoscopic ultrasonography with fine-needle aspiration, integrated positron emission tomography with computed tomography, and computed tomography in restaging patients with esophageal cancer after neoadjuvant chemoradiotherapy. J Thorac Cardiovasc Surg.

[13] Mesenas S, Vu C, McStay M, Forshaw M, Doig L, Mason R, Boyle N, Meenan J (2008). A large series, resection controlled study to assess the value of radial EUS in restaging gastroesophageal cancer following neoadjuvant chemotherapy. Dis Esophagus.

[14] van Rossum PSN, van Lier ALHMW, Lips IM, Meijer GJ, Reerink O, van Vulpen M, Lam MGEH, van Hillegersberg R, Ruurda JP (2015). Imaging of oesophageal cancer with FDG-PET/CT and MRI. Clin Radiol.

[15] Hong SJ, Kim TJ, Nam KB, Lee IS, Yang HC, Cho S, Kim K, Jheon S, Lee KW (2014). New TNM staging system for esophageal cancer: what chest radiologists need to know. Radiographics.

[16] Gollub MJ, Prowda JC (1999). Primary melanoma of the esophagus: radiologic and clinical findings in six patients. Radiology.

[17] Wong VK, Lubner MG, Menias CO, Mellnick VM, Kennedy TA, Bhalla S, Pickhardt PJ (2017). Clinical and imaging features of noncutaneous melanoma. AJR Am J Roentgenol.

[18] Tang Y, Jiang M, Hu X, Chen C, Huang Q (2021). Difficulties encountered in the diagnosis of primary esophageal malignant melanoma by 18F-fluorodeoxyglucose positron emission tomography/computed tomography: a case report. Ann Palliat Med.

[19] Cheung MC, Perez EA, Molina MA, Jin X, Gutierrez JC, Franceschi D, Livingstone AS, Koniaris LG (2008). Defining the role of surgery for primary gastrointestinal tract melanoma. J Gastrointest Surg.

[20] Wang S, Tachimori Y, Hokamura N, Igaki H, Kishino T, Kushima R (2013). Diagnosis and surgical outcomes for primary malignant melanoma of the esophagus: a single-center experience. Ann Thorac Surg.

[21] Dai L, Wang ZM, Xue ZQ, He M, Yuan Y, Shang XQ, Chen KN (2020). Results of surgical treatment for primary malignant melanoma of the esophagus: a multicenter retrospective study. J Thorac Cardiovasc Surg.

[22] Tan Y, Xiao EH (2013). Rare hepatic malignant tumors: dynamic CT, MRI, and clinicopathologic features: with analysis of 54 cases and review of the literature. Abdom Imaging.

[23] Wang J, Yang F, Ao WQ, Liu C, Zhang WM, Xu FY (2019). Primary gastric melanoma: a case report with imaging findings and 5-year follow-up. World J Gastroenterol.

[24] Jiang W, Rice TW, Goldblum JR (2013). Esophageal leiomyoma: experience from a single institution. Dis Esophagus.

[25] Elbawab H, AlOtaibi AF, Binammar AA, Boumarah DN, AlHarbi TM, AlReshaid FT, AlGhamdi ZM (2021). Giant esophageal leiomyoma: diagnostic and therapeutic challenges. Am J Case Rep.

