# Supplementary figures and images for: Primary malignant melanoma of the esophagus: differentiation from esophageal squamous cell carcinoma and leiomyoma using dynamic contrast-enhanced CT findings
Source: Abdom Radiol (NY). 2022 Jun 6;47(8):2747–59. doi: 10.1007/s00261-022-03556-8 (PMC9300547; doi:10.1007/s00261-022-03556-8)

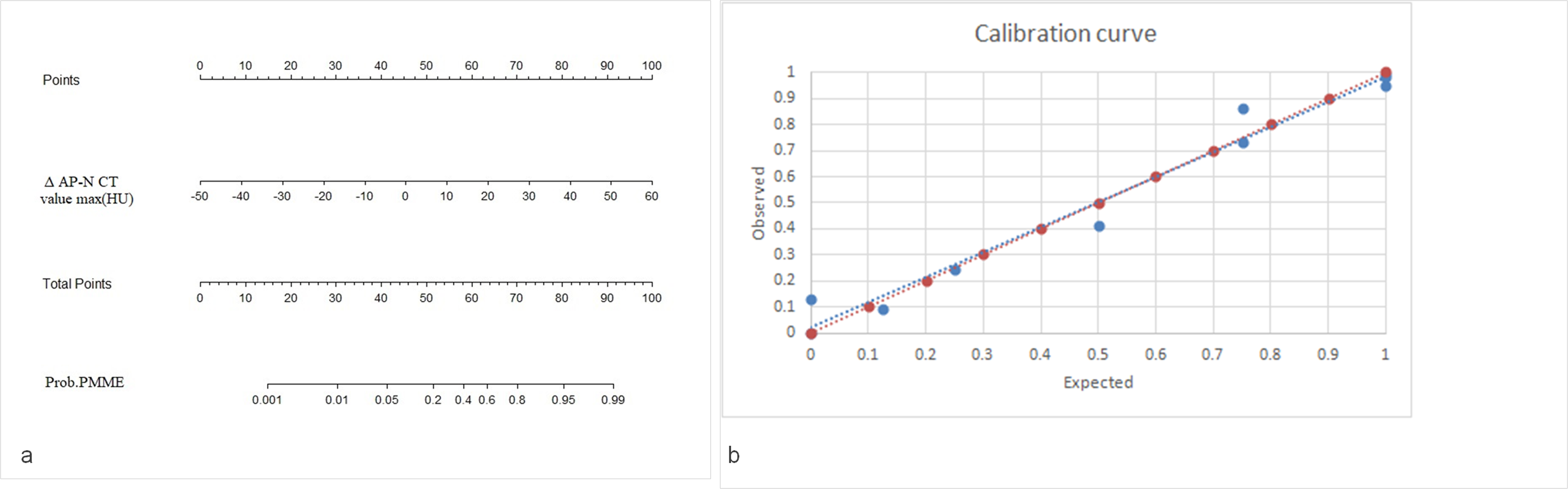

Supplement: Supplementary file 1 — Supplementary file1 (TIF 1710 KB) Fig. S1 Nomogram based on ΔAP-N CT value max for differentiating PMME from esophageal leiomyoma and the corresponding calibration curves. (a) The developed nomogram. (b) Calibration curves depicted the calibration of nomogram in terms of the agreement between the predicted probability of PMME and actual outcomes of the PMME. The y axis represented the actual probability of PMME. The x axis represented the predicted probability of PMME. The red line represented a perfect prediction by an ideal model. The blue line showed the performance of the CT model. The blue line was closer to the red line, which suggested a better prediction. [file 261_2022_3556_MOESM1_ESM.tif]
